# ‘The Second Arrow’: A Collaborative Autoethnographic Exploration of What Can Be Learned From One Long COVID Journey

**DOI:** 10.1111/hex.70227

**Published:** 2025-04-23

**Authors:** Sarahjane Belton, Kate Sheridan

**Affiliations:** ^1^ School of Health and Human Performance Dublin City University Dublin Ireland

**Keywords:** chronic illness, interdisciplinary care, psychologically informed practice, recovery, rehabilitation

## Abstract

**Introduction:**

Long COVID is a complex multisystem illness with multiple relapsing‐remitting symptoms, which can vary in severity and impact people's daily lives. This study utilises the first author's experience of falling ill with and recovering from long COVID to investigate the lived experience of the illness. Learnings that could positively influence how people with long COVID, and health professionals, approach rehabilitation and recovery from the illness going forward are identified.

**Methods:**

Employing collaborative autoethnography, the first author investigated her personal experience of falling ill with, and rehabilitating from, long COVID, while soliciting input of the second author (an athletic therapist and physiotherapist, and researcher with expertise in chronic pain) for the purpose of analysis and interpretation. Reflexive thematic analysis was employed across a number of data sources available to the first author, including journal entries, text messages, emails, and pharmacy receipts.

**Results:**

Four themes were generated from the data, supported by a number of subthemes: (i) Psychosocial impact of long COVID, (ii) Invalidated, (iii) Validated, and (iv) Power and Ownership. The negative impact of a siloed and reductionist approach to care for long COVID is evident in the findings of this study. In addition, the need for healthcare environments that enhance autonomy and empowerment, and that implement patient‐centred care, where the person living with chronic illness is supported to engage in management strategies that meet their needs, is underlined.

**Conclusion:**

This study highlights the detrimental cost, both personally and financially, of the ongoing use of the biomedical model of care in the treatment of long COVID. Findings support the need for an interdisciplinary approach to care that considers the whole person and adopts a biopsychosocial approach to care. Furthermore, the need for healthcare professionals to actively listen to, respect, validate and support the person living with long COVID on their individualised recovery journey is evident.

**Patient or Public Contribution:**

The first author was a long COVID patient, the context and extent of this is explained within the paper. As such, this paper is developed and written primarily from the perspective of a patient, as a first‐hand narrative of the recovery journey from the illness, with the insights of a clinician (second author) providing context and the potential for a broader understanding of the journey. The goal of this work is, through the dissemination of the paper's findings, to improve pathways and outcomes for others living with long COVID.

## 1 Introduction

Long COVID (LC) has been characterised as ‘a confusing illness with many, varied and often relapsing‐remitting symptoms and uncertain prognosis’ [1]. Many challenges are faced by persons with LC in receiving support for management and treatment of their condition, with the uncertainty and lack of knowledge around the condition within the medical field at the core of many of these challenges. Difficulties reported include accessing healthcare, not being taken seriously, receiving a diagnosis, disjointed and siloed healthcare, and large variation in quality of therapeutic relationships [1, 2].

The symptoms and pathomechanisms of LC show considerable overlap with myalgic encephalomyelitis/chronic fatigue syndrome (ME/CFS) [3, 4], as well as other persisting illnesses that can follow a range of other infectious agents and major traumatic injury [3]. Dysfunction of the autonomic nervous system (ANS) has been identified as a critical aspect of ME/CFS [5, 6, 7], with autonomic impairment also considered to play a central role in the underlying pathophysiology of LC [8, 9, 10]. Pharmacological intervention for ME/CFS has been shown to lack efficacy for many, and psychological interventions focussed on self‐management skills to improve symptoms and daily function have been developed [11, 12].

While the direct impact of LC on daily activity and quality of life is clearly evident, the relapsing‐remitting nature of LC has perhaps less tangible but more significant long‐term effects on both the personal identity of the sufferer and their recovery [13]. The need for multidisciplinary healthcare in addressing the challenges faced by persons with LC has been highlighted [1, 13, 14, 15]. Additionally, it is recognised that with conditions such as LC, where evidence is only emerging, people need to take the lead in finding solutions to their problems and accessing their own care [14, 15, 16].

Within the context of LC as outlined above, this paper details the lived experience of the first author, from the time point of falling ill in 2022, to regaining independence in 2024. The aim of this paper is to outline the barriers experienced to rehabilitation, and the key factors which supported successful rehabilitation from LC.

## 2 Methods

Autoethnography (AE) is an extension of ethnography, whereby the researcher is also the participant, and they position their own experiences as the key data to be understood; ‘Autoethnography is an interprevist and inductive research method that does not aim to produce reproducible truths about the world, but rather examines a particular phenomenon through the lens of the researcher's body and subjectivity’ [17, p. 3]. AE starts with a personal story, in a way that invites personal connection rather than analysis, but explicitly links the personal narrative to concepts from the literature [18]; using ‘personal experience to make unique and unfamiliar aspects of social life familiar to insiders (i.e. those with similar experiences) and outsiders (i.e. those without a shared point of reference)’ [19, p. 3].

The challenges the first author faced in employing AE, was ensuring that the narrative discussion was explicitly linked and situated within current research related to chronic illness [18], whilst also ensuring that the key elements that emerged were accessible and familiar to ‘outsiders’ [19]. Collaborative autoethnography (CAE) is a process, which involves collaborators at varying levels of participation [20]. Researchers employing CAE work together to analyse and interpret AE data to create a shared understanding [21]. Employing CAE in the current study allowed the first author (a researcher with expertise in health literacy, physical literacy, and physical education) to document her personal experience of falling ill with, and recovering from, LC, while soliciting input of the second author (an athletic therapist and physiotherapist, and researcher with expertise in chronic pain) for the purpose of analysis and interpretation [19, 20].

The person‐centred lens adopted allowed us to investigate the first authors human experience within the chronic illness landscape [22]. Researchers using ethnography typically align with social constructivism and interpretivism [22]; this is true also for our author team. The first author, in particular, both influenced and actively constructed the collection and selection of data. The interpretation of data is influenced by the first author's personal experience with chronic illness and experience as a researcher, and as such is a unique interpretation. Meanings are negotiated within the particular contexts of both authors, and as such is unique to our interpretations [23].

Within this CAE, we employed a mosaic approach (combining qualitative and quantitative data sources) to allow a broad awareness of multiple truths [22], to explore the practical and very real struggles the first author experienced navigating the effects of LC within and beyond the clinical setting. The collaborative approach allowed the first author's first‐hand familiarity with the experience (and data), and the second author's observation of the experience, and clinical and research expertise in the field of chronic pain and illness, to be combined [19]

our approach here may be especially important in relation to understanding the social construction of health and illness in people's lives because it allows an outsider to witness and examine the, often deeply private, health‐related experiences, emotions and thoughts of another during a time of interpersonal, emotional and cognitive change.

[19, p. 726]

### 2.1 Research Question

What can be learned from the first author's experience that could positively influence how people with LC, and health professionals, approach rehabilitation and recovery from the illness?

### 2.2 Data Sources

I (first author) collated the data for this paper from a number of personal sources; see Table 1.

**Table 1:** Data sources.

Source	Timeframe
Short descriptive notes made on phone ‘notes’ app [Phone Notes]	Months 1–5
Daily entries in an activity journal, kept to track daily movement, tasks completed and step count [Activity Journal]	Months 4–11
Journal entries reflecting lived experience [Journal]	Months 5–18
Text messages sent to close friends [Text]	Months 1–18
Email threads regarding medical appointments and treatments [Email]	Months 3–18
Medical documentation, including receipts and written communications	Months 1–18

### 2.3 Data Analysis

We used reflexive thematic analysis [24, 25, 26] as the primary method to analyse the data gathered, with a retrospective approach taken to working with the materials referenced in Table 1. Consistent with others [27], this is a technique that both authors had utilised separately in previous research and had some expertise in employing. Data analysis involved identifying key excerpts from my experience as captured in the various data sources and presenting them as a diverse range of codes (see Supporting Information for further information). We subsequently met to discuss these codes, during which meeting, the second author acted as a critical friend [28]. Some suggested wording changes were made, reflecting terminology routinely used in the literature. Where such changes made sense, and we both felt that the wording accurately captured the meaning, the change was made. This same process was then applied to the development of subthemes and themes until a comprehensive, coherent, and representative set of results were finalised.

I drafted the initial results/discussion based on the set of themes and subthemes, explaining and describing meaning, and presenting examples from the data set to support the discussion. I shared this with the second author, who made preliminary notes in terms of how what was presented linked to current literature in this area. Following this step, we collaboratively went through the results/discussion in an iterative fashion, via dialogue back and forth, refining the overall messages and learnings for rehabilitation and recovery from LC.

### 2.4 Context of the Paper

This experience is set within the Republic of Ireland, which has a mixed public and private healthcare system. I fell ill with COVID‐19 in June 2022. On Day 3 of the illness, I was struck very suddenly with a very severe headache, so that I could not remain upright. My description at the time was that it felt ‘like my head had been split open with an axe’ [Text]. This headache was constant from the time of its onset. On Day 5 this triggered a migraine, and from that point onwards I fluctuated between a severe headache and debilitating migraine (a new onset of persistent pain), with no relief in between. As the other ‘normal’ flu‐like symptoms of COVID gradually disappeared over the following fortnight, migraines and headaches, along with nausea, altered taste and smell, severe light and sound sensitivity, and dizziness remained. My sleep became severely disrupted (1–2 h in a 24 h period). I sought treatment initially from my General Practitioner (GP), and a vestibular rehabilitation physiotherapy specialist, but my condition continued to deteriorate. After 2 weeks I attempted a return to work, but a short period (7 min) behind a home computer triggered a migraine which lasted 48 h. My situation 9 weeks into this illness, is captured below:

… I had lost 5 kg, had a migraine constantly for 5 days in a row, was constantly nauseated, was sleeping 1–2 h a night, was floored by constant tiredness, and struggled to speak. If I was asked a question that required me to make a decision I couldn't even process it, let alone respond.

[Journal]

I was admitted to a private hospital, under medical insurance, 9 weeks following symptom onset, and remained there for 5 weeks. My ability to function deteriorated significantly over this time. Sitting in an upright position for more than 5 min resulted in severe pain with intense nausea. Completing independent self‐care such as walking the short distance to the bathroom became extremely difficult. Despite a significant daily effort to consume high protein and calorie food, my weight was reduced by a further 5 kg. My heart rate would elevate significantly, coupled with dizziness, with the effort to move from lying to sitting, and to a greater extent from sitting to standing. Despite the introduction of various medications, pain remained consistently high (between a 7 and 10 out of 10), and sleep was limited to 2–3 h/night. The below extract captures my experience in hospital:

I could barely move in the hospital room, I couldn't listen to music, watch TV; even sending or reading a text message was a huge challenge. None of the medication they tried, including opioids such as oxycontin, had any impact on the pain. I lay in bed with no energy and severe pain day and night, with icepacks wrapped around the front, back and sides of my head to try alleviate some of the pain. As the ice packs melted, my pain increased in severity.

[Journal]

While an inpatient, I was assessed by a general medicine physician, two neurologists, a cardiologist, and a pain specialist. In Weeks 4 and 5, at the insistence of a friend to the medical staff, I was also visited by a physiotherapist and dietician. An infectious disease consultant and an occupational therapist were requested, but the hospital could not comply. I underwent three brain MRI scans, chest X‐ray, brain CT, ECG, and echocardiogram, along with a range of blood tests. All tests returned clear. In an effort to control the migraine pain, I was prescribed a 5‐day infusion of Dihydroergotamine (DHE), which failed to have an impact. Each new specialist prescribed additional medication. When I left hospital, my prescription included 12 different items, as shown in Table 2. The medical bill associated with this hospital stay was €40,271 [Health Insurance Charge and Benefit Statement, 08/11/2022].

**Table 2:** Pharmacy prescription 20 September 2022.

Stemetil 5 mg
Pizotifen 0.5 mg
Sumatriptan 50 mg
Rivotril 0.5 mg
Venlaflex 75 mg
Topomax 100 mg
Esomeprazole Actavis 20 mg
Astillan 10 mg
Provera 10 mg
Testogel 50 mg
Molaxole Powder
Paracetamol 500 mg

**Figure 1:**
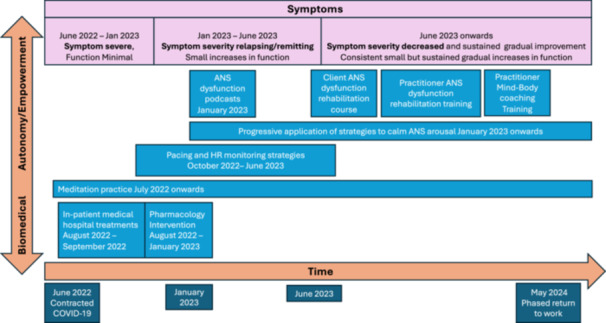
Overview of illness and recovery timeline.

Following discharge ‘[my husband] sought support from a community occupational therapist, but was declined on the grounds that I was “too young” [Journal]’. I ceased seeking help through traditional medical routes at that point and minimised interactions with medical professionals. I remained almost exclusively at home for a year, moving between a bed, floor, and sofa. Following this, I was slowly able to start exercising in a more meaningful way and resume some basic family and household roles. At this point, I regained some capacity for screen use, and started to research to try understand the illness and identify and implement appropriate tools and strategies to aid recovery. My narrative upon the themes which generated from the data in this study is influenced by this research. Over the course of the second year of the illness, my confines gradually expanded to include other quiet environments, such as local parks or forests, nearby friends' houses, or visiting family. My ‘symptomatic’ experience of LC shifted and changed over the 2 years. For example, as migraines started to reduce in duration, frequency and severity; anxiety, fatigue, and post‐exertional malaise emerged as predominant and rendered normal daily living very difficult. I was unable to work for 2 years due to the impact of long COVID. After 23 months, as part of my rehabilitation plan, I began a slow‐phased return to work – at which point this paper was initiated. Figure 1 presents an overview of my experience, developed to visually present the timeline and capture the various phases of my recovery journey.

## 3 Results and Discussion

**Figure 2:**
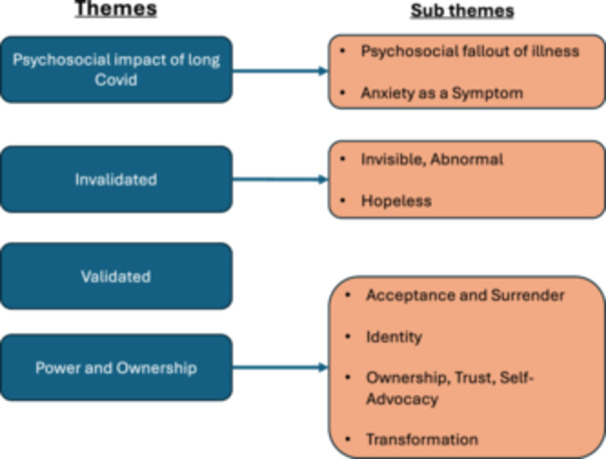
Study themes and subthemes.

Themes and subthemes are presented in Figure 2 (further detail including codes provided in the Supporting Information) and discussed under the following subsections, with discussion points threaded throughout. Where the first‐person narrative is employed, it captures the personal experience of the first author, along with how the first author has understood and interpreted her experience within literature.

### 3.1 Theme 1: Psychosocial Impact of Long COVID

#### 3.1.1 Psychological Fallout of Illness

A year into this illness, these are the words I used to describe the experience:

… falling ill with LC was like suddenly landing trapped inside a dark cave, wrapped in layers upon layers of dark rock, but not knowing that that was where I was. I could see nothing. I had been abducted, unceremoniously plucked from my life, and landed there with neither explanation for why, nor instructions for getting out. It was dark and compressing, foreboding, lonely, and isolating. It was also terrifying and confusing.

[Journal]

Many authors have discussed the significant role that shame plays in the experience of chronic illness [29, 30, 31, 32, 33]. I did not realise until many months into the illness that shame was something that had impacted me hugely, giving rise and intensity to, and of course, interdependent with, many other psychological impacts.

Shame — the deep sense of not belonging, of being defective or deficient in some way… is a painful and pervasive social emotion that also involves our thinking processes and sense of self‐worth.

[29, p. 126]

An initial response of isolating ourselves is common for many people living with a chronic illness [33], in a society where health is valued, and not being healthy is viewed as being inadequate and weak [33]; shameful. The negative link between the high levels of shame experienced by persons suffering from chronic illness, and psychological health has been highlighted [32].

In my experience, shame is derived from a number of sources. First, the shame of being so ill from a virus that others, anecdotally, seemed minimally affected by:

The narrative around COVID in our society, by the time I caught it, had moved from one of abject fear to one of cool nonchalance, ‘no big deal, get on with life, it's just a head cold’. So of course when I caught it I just assumed I would be able to row in with that narrative…. How could I be so sick, so incapacitated from a virus that everyone else seemed to recover from after a few days?

[Journal]

Along with broader society, the attitude of many in my closer social and family circle also fuelled this narrative. Although I had the unwavering and compassionate support of a tight‐knit immediate family group, and very close friends, text messages and comments from other family members, friends and co‐workers perpetuated the narrative of ‘surprise and disbelief’ that I was still sick; ‘I presumed you'd be better by now’ it was ‘just COVID’ after all [Journal].

Not being able to return to work was a major source of shame, with societal expectations of ‘normal’ recovery from illness prevalent; ‘I had the same expectations of myself as others did of me; Illness is weakness. Get over it, get on with it, get back to work’ [Journal]. Comments such as ‘so when are you going back to work?’, or ‘are you still not back at work?’, or ‘you really haven't worked in a year?’ [Journal entries] were not uncommon. While I received truly supportive and kind messages from many people across the hierarchy at my workplace, some isolated interactions were very difficult. For example, despite up to date medical certification of illness, and being unable to sit upright for more than 30 min at the time, my (then) senior faculty academic manager advised I would forgo a promotion opportunity if I

decided not to attend the interview the following week…and would need to prove when I returned to work that I was still performing at the same standard. These words floored me. I can't fully express the effect. In the first instance, the suggestion that being ill and unable to work was a ‘decision’ I was making was one of the most damaging things that anyone had expressed to me during the illness. But secondly, the articulation that I was no longer considered of any value to the institution gutted me…There was a lot of anger of course, but most overwhelmingly it was shame.

[Journal]

My experience in hospital also fed significantly into the experience of shame. Different consultants iteratively commenced and ceased treatment, with a narrative that I was abnormal and had failed to respond to their treatment. The majority of non‐consultant medical staff were kind and empathetic, however some interactions were less so, with statements from nurses such as ‘A young one like yourself shouldn't be lying in that bed all day, I bet you've never had as much rest in your life’ [Phone notes entries]. This is consistent with reports that people with unexplained or poorly understood illnesses can face disrespect in clinical encounters [31], in contrast to recommendations that early stages of interventions should focus on the prevention of shame, stigma and social invalidation [34].

I engaged at regular intervals with Occupational Health (OH) physicians assigned by my employer. Before my phased return to work, these interactions were very often condescending and shaming. One OH physician coughed and joked ‘look, maybe I'm getting long COVID now too!’. Others noted that ‘returning to work following long COVID was no different to returning from maternity leave’, and that ‘returning at a level below 50% of my previous work level was far below expectations’ [Journal entries]. I insisted on 20% as a starting point as I knew that loading beyond this would cause a relapse; I was supported in this by a GP and an occupational rehabilitation physiotherapist. Given the obvious symptoms exhibited of post‐exertional malaise, an abnormal physiological response to both physical and cognitive exertion [35], and a common persistent symptom of COVID‐19 [36, 37], an individualised return to work approach focusing on rest and energy pacing is what should have been recommended and supported [38, 39]. Indeed, to prevent relapses graded exercise programs, such as starting at 50% of previous work level, is contraindicated in cases of post‐exertional malaise [40, 41]. I was fortunate to be connected with two OH clinicians from 24 months onwards, who were more supportive of this phased return to work approach.

Shame for me always came back to the sense that, despite how hard I knew I was trying, I was not good enough; I should not be sick. I felt completely trapped within the situation, and powerless to change it. Brown [30] supports this experience, stating that when you feel intense shame, you feel fearful, trapped, and powerless, and talks about the ‘by products of shame’ being fear, blame and disconnection. Fear and oftentimes terror (of pain and its impact) were constant companions. For over a year, when I was caught in an endless cycle of pain and other debilitating symptoms and struggled to get control of the situation; the emotions of fear, sadness and isolation were constant and unrelenting. I was so unwell that I locked myself away, and my world became very small, trapped by the illness:

I struggled to hold a conversation with people. It was like all of my senses were out of whack – light, sound, busy places in terms of noise or movement, even smells – all of these could trigger sickening headaches, if not a 12‐48 h migraine. I felt completely trapped, isolated, alone. And useless. Emotionally I was angry, confused, sad, terrified, and very very ashamed.

[Journal]

As I started to make some small ground in terms of recovery, I developed sufficient perspective to see, understand, and interpret this emotional experience. ‘Identity’ emerged at this point as central to my understanding. Before falling ill, I had been a wife, mother to a 12‐year‐old, full‐time academic and researcher, volunteer coach, and an active and healthy individual. These roles had formed ‘identities’ for who I thought I was, and were unceremoniously ripped from me when I fell ill with COVID. This loss of identity, and change in self, is in keeping with previous authors' documentation of the impact of living with chronic pain [42], and the impacts underline the fundamental human need to belong and participate in society [43].

#### 3.1.2 Anxiety as a Symptom of Long COVID

Fear was a core aspect of my LC experience from onset; however, anxiety first became apparent 5 months later, as a very physical manifestation – in the form of severe pain, weight and tightness in my chest, and a palpable urge to ‘flee’.

It manifested almost exclusively as a violent physical sensation; thoughts didn't get too much of a look in it would happen so quickly…the brains way of communicating with me – ‘I feel incredibly unsafe, get me out of here now’.

[Journal]

The onset of anxiety coincided with my first outing from home following hospital. From this point onwards, the physical sensation, and associated psychological imperative to flee my environs and context, were ever present, varying only in degree of severity. As migraines slowly decreased in intensity, frequency and duration, this anxiety increased – until it became a new debilitating symptom of LC, following the same pathways as pain, fatigue, dizziness, nausea and the other symptoms. This aligns with the perspective on chronic symptoms being a result of neural circuit disorders, where an initial structural or physical cause for symptoms has resolved (e.g., the COVID‐19 virus), but symptoms are perpetuated by learned neural circuits in the brain [44, 45, 46]. Within this perspective, symptoms such as migraine, headache and dizziness, are considered equivalent to others including anxiety [45].

### 3.2 Theme 2: Invalidated

I tried multiple times while in there to advocate for myself, but hadn't the strength, in fact often I struggled to retrieve words from my brain as I needed them. I knew that the fact that they were all treating my case by looking only at individual symptoms was an issue. Once I did manage to say to my main consultant ‘This is long COVID, this isn't a migraine issue, or a blood pressure issue, or a breathing issue. It's all been caused by COVID, that's the root cause, how are we treating that? Can we not at least bring in an infectious disease specialist?’. He retorted ‘what would he know?’

[Journal]

This theme is largely reflective of my interactions with medical professionals, primarily within but also beyond hospital. While it reflects my experience with a majority of medical professionals, I underline that it is not reflective of all; there were also many instances of compassionate kindness.

#### 3.2.1 Invisible, Abnormal

I never saw those neurologists again. They couldn't help me, so I ceased to exist in their world – not their problem. That was soul destroying. I had entered the hospital full of hope, and all of their best minds came to help, but when they couldn't figure things out, one by one they just disappeared.

[Phone Notes]

As described under Theme 1, many consultants reviewed my case, prescribed medication, and when no improvements were made, they disappeared; my case was wordlessly dismissed. The concept of ‘Medical Invalidation’ matches this experience;

Medical invalidation, whereby health care professionals dismiss, minimize or otherwise do not take patient concerns seriously, is a well‐documented phenomenon in the literature on chronic illnesses.

[47, p. E915]

Specific to chronic pain, pain invalidation is reported as a sense of being disbelieved when no organic cause for pain can be found [48]. I felt invisible and inaudible in hospital, treated as a set of independent symptoms by a range of different consultants, rather than a whole person who was experiencing all of these symptoms. This experience is reflective of others with LC also and aligns with the concept of ‘reductionism.’

When long COVID patients seek medical care, medical treatment technology reduces the “lived body” into an object body, the patient's experience of illness is less important compared to medical tests and imaging indications, and conclusive diagnosis is made with the help of sophisticated medical equipment and data. The unverifiable subjective experience renders even modern medicine helpless, exacerbating the anxiety of long COVID patients.

[49, p. 5]

Efforts to advocate for myself continually failed, I often struggled to retrieve words I needed to use. A close friend accessed the consultant and demanded their attention, and advocated on my behalf; however, the response was to continue to prescribe medication for each symptom individually; and my condition continued to worsen.

They could not (or would not) look at the big picture – they just were not trained to take in the whole human in front of them… The result was that their treatment, and messages to me, made things a lot worse.

[Journal]

The collective impact of medical invalidation and reductionism was that I felt completely disempowered. I was reduced to a set of symptoms, unworthy of a conversation from consultants around me; 2–3 days between consultant visits became normative as an inpatient. Whenever I raised the point that I had been on the medication for increasing numbers of weeks and that my condition had worsened considerably over that time, I was dismissed repeatedly with a patronising air ‘you have to give my medication time to work’ [Phone Notes]. The message ultimately changed over the 5‐week hospital stay to ‘let's just get you well enough to get you home, you are going to have to live like this’ [Phone Notes].

#### 3.2.2 Hopeless

In contrast to the integral recommendations that healthcare professionals should actively listen, believe and treat the person in pain with dignity [50], in hospital, I felt inaudible and abnormal. The tone and words of the consultant made it clear that there was no flaw in their treatment plan; I was simply failing to respond to it, failing to recover. This experience of critical judgement and blame, relating to invalidation at the level of self, is prevalent in research [34].

I felt abnormal, unwanted, in the way, and beyond help. With my body unable to respond the way it was ‘supposed to’ I felt completely isolated and hopeless. I also felt a growing sense of discomfort from many of the nursing staff as they came in and out of my room. They could see how much pain I was in but couldn't do anything to help, and the answer from many was to avoid my room as much as possible. I understood fully. If I could have avoided myself in any way I would have too.

[Journal]

I left the hospital confused as to why my body was behaving the way it was, and with a deep fear compounded by the knowledge that after 5 weeks, all medical personnel had been unable to help me. At that point, my situation felt completely hopeless with nowhere left to turn for help [51], and I felt let down and alone [16]. My final interaction with the hospital consultant, 2 months after my discharge, is captured in this Journal entry; *‘*In that visit he told me that I was going to end up back in hospital because I had weaned myself off “his medication”. His tone and manner were that he was wiping his hands of me*’*. I knew that the medication had not helped and had negatively impacted my usual reserve of mental strength and resilience. This final interaction solidified a resolve to manage my own recovery from that point onwards.

It was highlighted early on that LC rehabilitation required a biopsychosocial approach to care, centred on improving a person's function, decreasing disability and supporting engagement in society [52, 53]. In contrast, the first author's experience describes a biomedical model of care, exemplified by discharge from the hospital with a prescription for 12 medications (indicative of excessive polypharmacy [54]), and with no referral for interdisciplinary care to support rehabilitation.

### 3.3 Theme 3: Validated

Although I encountered significant negative, dismissive and disempowering interactions with medical personnel, I was fortunate to have positive, supportive, and helpful interactions with other health professionals. The first of these was my co‐author, a friend and colleague with considerable experience working with people rehabilitating from chronic illness. A significant step in validating a pain experience involves advising the person in pain that they are not alone nor abnormal [48]; this health professional elucidated that others were suffering similarly from LC and provided continuous reassurance that I would recover. Pacing, a conscious effort to regulate activity to avoid post‐exertional malaise, is a prominent strategy applied in Chronic Fatigue Syndrome and post‐viral disorders [55]. She provided practical advice regarding paced activity, which over time allowed me to gradually increase function. Her advice normalised a recovery pathway that would have many peaks and troughs, and so when I experienced relapsing episodes, I was able to hold perspective and maintain progress. Critically, when I suffered significant setbacks, her advice helped me to accept it, allow time for my body to ‘get back on track’ and then continue my journey.

Don't try to walk, or swim, or go for coffee [she said], just let it all go for now and rest up – treat it like a tapering week/fortnight like you would have when you were training for Rugby. This made a massive difference, and things slowly started to shift once I just let go.

[Activity diary]

The second critical support I received was from another physiotherapist and athletic therapist good friend and colleague. He provided regular reassurance of the inevitability of recovery, along with routine treatments which targeted regulating my central nervous system. He highlighted similarities between my symptomology and those in athletes with post‐concussion syndrome (PCS). He explained that ANS dysfunction is considered one of the factors contributing to symptoms experienced in mild traumatic brain injury, including headaches, sleep disturbances, anxiety, cognitive impairment, and mood disorders [56, 57]. He suggested applying a similar rehab approach to that prescribed for PCS, monitoring heart rate, and as it spiked slowing down the rate, intensity, and pace of any activity I was engaged in. This approach, combined with the pacing advice, meant that very gradually over several months I was able to increase my physical activity.

A third health professional, a rehabilitation physiotherapist, later supported the approaches advised by my two colleagues, and further normalised my experience and recovery pathway. The critical impact of the interactions with these health professionals was that I did not feel alone and developed hope for recovery. The parallel drawn with PCS led me to research the syndrome and uncover a wealth of critical information in the field of neuroplasticity as it relates to chronic pain and illnesses including LC. This information further validated my experience. I understood that I had not failed the medical treatment; I had simply not received the treatment that I needed. Understanding the role that the brain, and specifically a constantly activated sympathetic response, was playing in my experience, was the corner stone upon which I built my recovery.

I was blown away by how central a role the emotion ‘fear’ played in perpetuating the cycle of pain – fear which I could now see wracked every facet of my body and mind, sleeping and waking, and was dictating my life.

[Journal]

About 6 months after hospital discharge, I was well enough to attend a consultation with an infectious disease specialist, which further validated my instincts and intuition regarding the illness, and my approach to recovery. *‘*He explained to me exactly what had happened to my body as a result of the COVID infection; that my brain was stuck in sympathetic activation, and that it was going to take time to come right. He explained that it wasn't fully understood how this was affecting the brain, nor did they yet have an effective treatment plan*’* [Journal]. When I spoke to this consultant about my experience with the hospital consultant regarding the medication, he expressed disbelief ‘but why would you keep taking them? It wasn't working!’ [Journal]. Research supports the positive impact that validation can have in recovery, helping individuals to view pain as less threatening [58] and reducing the grieving associated with the loss of former self [59]. Moreover, as was my personal experience, it provides a very clear sense that you are seen, and heard, and understood, which in turn provides a critical foundation upon which to develop the confidence and agency needed to recover.

### 3.4 Theme 4: Power and Ownership

The interaction with the infectious disease consultant provided what I hadn't realised I needed; utter confidence that the recovery approach and programme I had developed was correct. This confidence, and trust in myself, became central to my recovery. I started exercising autonomy and advocating for myself. I sought out specific help from health professionals when I knew what I needed. I trusted myself and my own growing body of knowledge and took full ownership of my recovery. I still met shaming experiences with health professionals but was able to quickly recover trust and confidence in myself and maintain my own recovery programme. In keeping with characteristics of self‐advocacy [60], the above highlights the critical role that enhancing illness education, assertiveness with healthcare professionals, and mindful non‐adherence played in recovery. Mindful non‐adherence, the ability to challenge treatments offered that do not meet the person's needs [60], was a key factor in progressing to self‐management and exploring mind‐body treatments.

#### 3.4.1 Acceptance and Surrender

It was rather like, having pushed as hard as possible with my shoulder against a door for months to try and open it, I finally surrendered and stepped back, only to realise that the door actually opened inwards.

[Journal]

As unlikely as it sounds, surrendering became a turning point in my recovery. This surrender was not a giving up, rather a full and unconditional acceptance of what was; ‘struggling with rather than against illness’ [61, p. 657]. Rather than fighting the pain and other symptoms, I started to welcome them, allow them, and almost befriend them. This act of ceasing to fight the symptoms, to fight for recovery, to fight against time that seemed to be running away from me, seemed to take my ANS by surprise, and allow it reduce the ‘fight or flight’ state that had perpetuated for over a year. My rate of recovery, though still slow, gradually increased, and the peaks and troughs started to even out. Acceptance is the readiness to experience pain, and the ability to participate in activities of daily life despite pain [62]. Identified as key in supporting function, pain acceptance has been associated with maintaining function [63], lower pain levels [62, 63], less pain‐related anxiety and depression [62], and lower risk of developing disability [63].

My approach to acceptance and surrender was to acknowledge where I was in the moment and accept fully what was present, always knowing, however, that it was *just* for right now [64], ‘The habit I slowly formed was to accept everything just the way it was, however unpleasant things were. I learned to acknowledge that it was unpleasant, … feel angry or sad or frustrated or scared, but accept things nonetheless as they were’ [Journal]. This practice of acceptance, or ‘detachment from outcomes’, is very similar to a corner stone pain reprocessing tool ‘outcome independence’ [46]. It also aligns with techniques of ‘mindfulness’ or ‘present moment awareness’ often advocated in recovery from chronic conditions [44, 46, 65].

#### 3.4.2 Identity

My contention is that illness, and especially chronic illness, is precisely that kind of experience where the structures of everyday life and the forms of knowledge which underpin them are disrupted. Chronic illness involves a recognition of the worlds of pain and suffering…which are normally only seen as distant possibilities or the plight of others.

[66, p. 169]

A popular Buddhist scripture guided much of my approach to recovery, ‘In life, we can't always control the first arrow. However, the second arrow is our reaction to the first. The second arrow is optional’ [67]. Though I experienced persistent and significant physical pain due to the various LC symptoms, the emotional suffering that I experienced was in large part down to loss of identity (see Theme 1). Learning to separate physical pain (the ‘first arrow’) from emotional suffering (the ‘second arrow’; related to loss of identity), which I accomplished through regular Zazen meditation practice (a new practice adopted early in the illness), was a core component of my recovery. This distinction is reflected in pain literature also, with pain and suffering recognised as inter‐related, but distinct experiences [68, 69].

The sensation of pain is intrinsically unpleasant, and this unpleasantness involves basic emotions. However, the more complex emotions, which can in turn enhance this unpleasant sensory and (basic) emotional experience, are not part of the pain sensation itself but of pain‐related suffering.

[68, p. 1445]

Suffering is understood as a subjective experience characterised by a negative affective valence, with disruption to one's sense of self an integral part of suffering [69]. I associate my ‘loss of identity’ when I could no longer carry out the many roles that had been central to my daily existence, with this use of the term ‘sense of self’ [69]. Identities are developed through the interaction between how we see ourselves, and how others see us [49]. Awareness and identification of bodily impacts, and resulting identity ‘trade‐off's’, is an important step in successfully adapting to living with impairment [61]. My recovery was fuelled by a direct intentional surrendering of prior identities, and ceasing to be defined by them. I realised also the danger of a new identity forming, that of a ‘sick person’, and made conscious efforts to ensure this new identity did not engrain; ‘Long COVID gives the patients the “sick role”, and it is imperative for them to regain their “healthy” social image and live as normal people’ [49, p. 7].

#### 3.4.3 Ownership, Trust, Self‐Advocacy

Self‐compassion, ‘the ability to remain kind and understanding of oneself in the face of negative external events’ [49, p. 8], was key in overcoming my psychological suffering around identity, which was linked with a deep sense of shame and blame I felt around the illness, and my perceived failure to recover. I recognised that I did not choose what had happened to me, I did not have conscious control over it, and it was not my fault [70, 71]. My experience in hospital, and following discharge, had forced me to surrender any expectation for medical assistance in my recovery. I listened to numerous audiobooks and podcasts [46, 71, 72, 73], and over time retrieved many read peer‐reviewed articles [74, 75, 76, 77, 78], which provided a background to the type of illness I was facing.

…by the time I stumbled across the term ‘fight or flight’, and started to uncover this treasure trail, I carried with me the gift of absolute desperation. I already knew I was on my own, and my future looked very bleak ‐ so when I found this information it was like finding an answer to my prayers.

[Journal]

Applying Schubiners critera [45], I self‐diagnosed a neural circuit disorder/ANS dysfunction [9, 12, 45, 57, 78], and identified tools to aid recovery. Meditation has already proven very helpful, and many of the strategies, learnings and tools I uncovered from my reading (audiobooks) in the space of eastern psychology and philosophy [64, 67, 79, 80], paralleled current neuroscience approaches. The various tools I employed (derived from a variety of sources, along with my own intuition), included meditation, developing skills of somatic awareness and somatic tracking during daily activities, befriending (rather than resisting) pain and unpleasant sensations, imaginal exposure and gradual re‐engagement to activities, ‘brain talk’ (constantly reassuring brain of safety), extended exhale breathwork and humming, self‐compassion practices (including meditation and journaling techniques), and an attitude of nonattachment to results (de‐coupling cause and effect, akin to ‘outcome independence’ [46]).

The development of self‐efficacy, belief in my capacity to execute behaviours necessary to recover from the illness [81], was critical. Within Bandura's theory [81], self‐efficacy is considered the critical agent of therapeutic change and has been associated with self‐management of chronic disease [82, 83, 84]. As I applied these various tools, I made gradual improvements in function and experienced gradual decreases in pain and other symptoms. I enrolled iteratively on self‐paced online certificate courses aligned to the area (total cost of training courses; €2312), which provided the multi‐benefits of (i) rehabilitating my tolerance for use of screens, (ii) rehabilitating my cognitive capabilities, (iii) enriching my knowledge and understanding in the field of neuroscience of chronic illness and (iv) learning new strategies which I could apply to my own recovery. An increased knowledge in pain neuroscience has been shown to support coping skills by decreasing the threat value of pain [85]. My confidence and trust in my own instincts and abilities grew, and I learned to self‐advocate. LC has been acknowledged as an illness where evidence is emerging, and as such ‘the patient has to take the lead in finding solutions to their problems and accessing their own care’ [16, p. 833]. When I recognised I had a particular need for assistance to aid my recovery, I would source someone or something to meet the specific need identified. I also took affirmative action to quickly cease any treatments or modalities when I knew they were not helpful and turn down suggested treatment avenues which did not align with my recovery approach.

#### 3.4.4 Transformation

Transformation is a common theme in the narrative of people living through a difficult illness; that something good eventually emerges from what at the time was the most difficult challenge they ever faced [16, 49, 51, 86]. As I reached the point of surrender and acceptance, this idea of transformation emerged within my own narrative:

… acceptance doesn't mean giving up either, it doesn't mean accepting this as my fate forever. It means accepting that just for right now my situation is what it is, and not trying to push it away… I could consider my ill luck as an opportunity. I was able to look at the situation as something that had been thrown across my path to help me somehow grow as a person, and as such every part of it, no matter how difficult, was exactly where I was meant to be at that moment.

[Journal]

Hope that some good would come from their experiences has similarly been expressed by others with LC [16], along with psychological growth, where people learn to view problems with gratitude, through the cherishing of life, family, bravery and tenacity [51]. The individual resilience and mental strength that people find in the face of illness, to be able to face everything in their lives with more optimism, has also been reported [49]. The enforced slow pace of life that LC insisted on, brought space and time to consider the purpose of life, and my role in it. My clinging to old identities ceased, and I found that I no longer wished to have my ‘old life’ back, but approached my recovery, and whatever lay ahead, with a surety that something meaningful would emerge;

I no longer lament the seeming harshness of what was ripped from me. Rather I appreciate that, while hugely difficult to navigate all the loss and suffering, now I have been offered a very rare opportunity to start again, a blank canvas.

[Journal]

## 4 Summary Discussion and Implications

Social construction of illness is heavily informed by the ways in which patients and clinicians define and label an illness [87]. Meaning is assigned to a set of symptoms, relating to existing medical understanding and hypotheses about a given set of biological phenomena [19]. A definition of the situation is made to guide action, the question ‘what is going on here?’ is asked, and a decision is made as to what should be done in response [19, 87].

When a given illness is framed accurately, the strategies prescribed may aid in the management of the condition over time. By the same token, an incorrect framing may have disastrous effects on the attempt to manage a given set of symptoms and necessitate a reframing – or reconstruction – of what the condition is and how to manage it. Indeed, the concept of accuracy in framing requires attention to multiple perspectives that may be relevant in analysing a social phenomenon such as chronic illness.

[19, p. 724]

This study underlines the critical importance of accurate framing, along with a deliberate effort early in chronic illness interventions to reduce shame, stigma and invalidation [34, 88]. By identifying individual beliefs and feelings associated with LC symptoms, the first author developed self‐management skills addressing the behavioural aspects of the symptoms, a process referred to as psychologically informed practice (PIP) [89]. Communication strategies employed by a healthcare professional can play a key role in the implementation of PIP [90]; however, poor communication and constant invalidation dominated early treatment interventions for the first author, resulting in a period of 12 months before she had the capacity to research PIP independently. This experience cannot be considered optimal care.

The first author's experience aligns with previous research highlighting the ongoing dominance of the biomedical model in relation to chronic pain [91, 92]. A patient‐centred approach, including a strong therapeutic alliance, is recommended in chronic illness rehabilitation [93]. Communication strategies such as openness, non‐judgement, listening and trust have been highlighted as key pillars to providing patient‐centred care [94]. Patient‐centred care is founded on the biopsychosocial model [95, 96], which considers the biological, psychological and social components of the ‘whole‐person’ and is in direct contrast to the reductionist biomedical approach experienced by the author [97]. In this case, the cost of the biomedical approach (for the hospital stay alone) was €40,271; the cost of her own biopsychosocial exploration of recovery amounted to €2312. The tables below present key recommendations from the perspective of a person with a lived experience of LC (Table 3, first author) and a clinician's perspective (Table 4, second author), which can inform a more positive experience for individuals and health professionals facing LC (and other chronic health conditions) in the future. Fundamentally, we recommend that healthcare professionals recognise the person is an expert in their own lived experience, and as such their voice must be heard, considered and integrated into any care plan. In particular, healthcare professionals should focus on achieving patient empowerment by supporting autonomy and competency in symptom management.

**Table 3:** Recommendations for people experiencing long COVID and other related chronic conditions.

Trust your own instincts, persist and insist on being heard
If not being listened to or respected, go elsewhere
Don't hand over all power to health professionals
If can't advocate for self, bring someone to consultations with you that can
Educate yourself, or seek out education, on the science behind chronic persistent symptoms
Accurately identify source/cause of symptoms, ensure treatment plans target the source, not just the various symptoms presenting
Engage with health professionals that validate your experience, can acknowledge that they may not fully understand the condition, are open to new scientific findings, and supporting new approaches to recovery
Progress at the pace that feels right for you, don't be pushed forward, or held back, by health professionals who do not understand the nature of the illness, or who are not actively listening to you
Recognise the role that emotional response can play in perpetuating symptoms, and consistently implement strategies to address this
Develop a personalised suite of tools, through engagement with variety of appropriate allied and other health professionals, to apply to your recovery

**Table 4:** Recommendations for health professionals working with people with long COVID and other related chronic conditions.

Actively listen to the person presenting with the chronic illness, they are the expert in their own symptoms
Adopt a whole‐person approach to care, ensuring a biopsychosocial model is implemented
Invite and welcome the patient as a shared partner in the healing process
Recognise that no one person or profession holds all knowledge or tools, and that an interdisciplinary approach to care is most likely to succeed
Validate the patient's experience, and understand the importance of preventing the onset of stigma or shame in relation to chronic illness
Ensure both you and the patient have an understanding of the neuroscience of persistent pain, chronic fatigue and other chronic symptoms. Use modern neuroscience to support the development of self‐management skills.

### 4.1 Strengths and Limitations

This paper reflects the first author's experiences and interpretation of the same, so generalisability to the experience of all those living with LC is not suggested, nor appropriate. It is also true, however, that the lived experience presented in this paper is not isolated or unique to the first author. The thorough triangulation and analysis of the breadth of data of this lived experience, offers a unique insight that could enhance rehabilitation and recovery for people with LC and other chronic illnesses.

## 5 Conclusion

This paper underlines the critical role of empowerment, self‐advocacy and validation in supporting successful rehabilitation from LC. The experience presented is one of not being believed, a lack of compassion, a lack of pain and symptom understanding, experiences of stigma, critical self‐judgement, a loss of identity and isolation; experiences that identically mirror the narrative of chronic illness invalidation presented in numerous other papers. This paper highlights a journey through an incorrect framing of the illness, through to the first author's own accurate framing. This accurate framing led to the identification of appropriate strategies to aid recovery, which proved a turning point in rehabilitation and recovery.

Findings underline the critical importance of a patient‐centred approach to care and recovery, along with the transformative impact of knowledge, validation and empowerment on such a recovery journey. We propose that the recommendations for health professionals presented in Table 4 should be adopted as essential actionable steps, and indeed as policy within health services, to improve the experience of, and outcomes for, people living with and recovering from chronic illness. In addition, in line with the recent work of Donnino et al. [44], the development, and investigation of the feasibility and effectiveness, of intervention strategies based upon the framing of LC as a neural circuit disorder (or ANS dysfunction), is warranted.

## Author Contributions

**Sarahjane Belton:** conceptualisation, methodology, data curation, formal analysis, project administration, writing – review and editing, writing – original draft, investigation. **Kate Sheridan:** formal analysis, writing – original draft, writing – review and editing.

## Ethics Statement

This autoethnographic exploration is based on an analysis of the first author's experience of living with and recovering from long COVID. The data sources collated and used in the analysis are derived from the first author's personal experiences and interactions on this journey with chronic illness. This experience was only identified 2 years after falling ill as an object of research. Consistent with other long COVID autoethnographic accounts, there was no planned research project, no prospective data collection, and no intention to perform research at the time the experiences and interactions occurred, and as such, obtaining ethical approval was not possible. The lead author acknowledges that studying oneself in this way involves a level of vulnerability but confirms consent in awareness and full consideration of that, that she is openly and willingly sharing here what she feels appropriate and necessary to potentially improve the trajectory of long COVID recovery for others struggling with the illness.

## Conflicts of Interest

The authors declare no conflicts of interest.

## Supporting information

Supporting information.

## Data Availability

The authors have nothing to report.
