# Supplementary material for: ‘The Second Arrow’: A Collaborative Autoethnographic Exploration of What Can Be Learned From One Long COVID Journey
Source: Health Expect. 2025 Apr 23;28(3):e70227. doi: 10.1111/hex.70227 (PMC12015975; doi:10.1111/hex.70227)
Supplement: Supplementary file 1 — Supporting information. [file HEX-28-e70227-s001.docx]

**Supplementary material**

**‘The second arrow’: A collaborative autoethnographic exploration of what can be learned from one long COVID journey**

**Data management, processing and analysis – additional information**

The data sources which informed this paper were collated by the first author from a range of personal sources. This data was managed and processed as follows:

- *Short descriptive notes made on phone ‘notes’ app*. These notes were taken from the Notes App and taken to an MS Excel file. Key excerpts were identified from these, and codes were assigned.
- *Daily entries in an activity journal, kept to track daily movement, tasks completed, and step count*. This was a hand written journal. Relevant information (including data such as step count, and key excerpts) from the journal was brought across to an MS Excel file. Key excerpts were identified from these, and codes were assigned, and important information (such as step count) which could support the excerpts were identified.
- *Journal entries reflecting lived experience*. This was a continuous MS Word document which the first author contributed to routinely from months 5 – 18 of her illness/recovery. Key excerpts were identified from these, and codes were assigned.
- *Text messages sent to close friends*. Relevant text messages were copied from phone, and pasted to an email. This text message content was then copied from email to an Excel file. Key excerpts were identified, and codes were assigned.
- *Email threads regarding medical appointments and treatments*. Relevant content from these were copied (or transcribed where only paper copies were available) to an Excel file. This information was later used to support themes and/or subthemes which were developed based on the other data sources.
- *Medical documentation, including receipts and written communications.* Relevant content from these were copied (or transcribed where only paper copies were available) to an Excel file. This information was later used to support themes and/or subthemes which were developed based on the other data sources

Supplemental Table 1 below shows the codes aligned with the paper sub themes and themes.

**Supplemental Table 1 Themes, Subthemes, and Codes**

| **Themes** | **Sub themes** | **Codes** |
| --- | --- | --- |
| **Psychosocial impact** | **Anxiety as a Symptom** | Anxiety |
| **of long covid** | **Psychosocial fallout** | Shame of illness |
|  | **of illness** | Fear and terror |
|  |  | Identity |
|  |  | Sadness and despair |
|  |  | Isolation |
| **Invalidated** | **Invisible, abnormal** | Dismissive |
|  |  | Disempowering |
|  |  | Reductionism |
|  | **Hopeless** | Abnormal |
|  |  | Not responding |
|  |  | Failure to meet expectations |
|  |  | Blame – ‘failure to recover’ |
|  |  | Uncertainty and confusion |
|  |  | Shame of not recovering |
|  |  | Nothing working |
|  |  | Learn to live with it |
|  |  | Noone can help |
|  |  | Being avoided |
|  |  | Hero to hopeless |
|  |  | Alone |
| Validated |  | Not alone |
|  |  | Normalised (others experiencing same) |
|  |  | Experts acknowledge lack of knowledge |
|  |  | Not alone |
|  |  | Seen as a whole person |
|  |  | Acknowledge cause of symptoms |
|  |  | Treat the whole person |
| Power and Ownership | **Acceptance and** | Surrendering, befriending, and welcoming |
|  | **Surrender** | Surrendering time |
|  |  | Letting go of expectation for medical |
|  |  | assistance in recovery |
|  | **Identity** | Self-compassion (letting go of shame) |
|  |  | Surrendering identity |
|  |  | Shedding the 'sick role' |
|  | **Ownership, Trust** | Autonomy and responsibility |
|  | **and Self-advocacy** | Finding my voice |
|  |  | Power of science |
|  |  | Knowledge and understanding |
|  |  | Trusting myself |
|  |  | Others trusting me |
|  |  | Ownership of recovery |
|  |  | Trusting my instincts |
|  | **Transformation** | Uncovering the gold |
